# Challenging diagnosis of Kimura disease in a child with nephrotic syndrome: A case report

**DOI:** 10.1002/ccr3.7031

**Published:** 2023-03-02

**Authors:** Roya Hemayati, Fariba Binesh, Mohammad Pashmchi, Seyyed Mohammad Kazem Mousavi Anary, Mohammad Mohammadi

**Affiliations:** ^1^ Department of Internal Medicine Shahid Sadoughi University of medical sciences Yazd Iran; ^2^ Department of Pathology Shahid Sadoughi University of medical sciences Yazd Iran; ^3^ School of Medicine Shahid Sadoughi University of Medical Sciences Yazd Iran

**Keywords:** focal segmental glomerular sclerosis, Kimura disease, methylprednisolone, nephrotic syndrome

## Abstract

Kimura disease (KD) is a rare condition with a challenging diagnosis because it may be misdiagnosed and not differentiated from other disorders. We reported a 13‐year‐old patient who presented with growing neck masses and was hospitalized due to the nephrotic syndrome relapse but was eventually diagnosed with KD.

## 1 BACKGROUND

Kimura disease (KD) is a rare, inflammatory, idiopathic condition characterized by painless, slow‐growing subcutaneous masses frequently in the head and neck region,^1^, ^2^ that occurs more commonly in young males with a peak incidence in their second to fourth decades of life.^3^

Renal involvement is the leading systemic manifestation of KD.^2^ Different types of kidney disorders have been reported to be related to KD, such as membranous glomerulonephritis, mesangial proliferative glomerulonephritis, minimal change disease, IgA nephropathy, focal segmental glomerular sclerosis (FSGS), and acute tubular injury.^4^ However, the underlying mechanisms of KD and its renal‐associated involvement remain unknown.^5^

KD diagnosis is often challenging and easily misdiagnosed due to its rarity.^6^

So far, different therapeutic approaches, such as surgical excision, immunosuppressive agents (such as thalidomide and cyclosporine), and local radiotherapy, are recommended for KD management.^7^, ^8^ However, there is still no specific protocol for definitive KD treatment, and KD patients experience a high rate of recurrence, up to 62%.^9^

Here, we reported a 13‐year‐old male patient who was hospitalized as a result of the nephrotic syndrome relapse and was eventually diagnosed as a case of KD.

## 2 CASE PRESENTATION

In September 2021, a 13‐year‐old boy presented to the emergency department of our medical facility complaining of fatigue and widespread edema over the last two months. Additionally, he reported two bilateral painless subcutaneous masses on both sides of his neck with progressive growth over the past six months.

He was previously admitted to our center in August 2020 with generalized edema and a preliminary diagnosis of nephrotic syndrome. At that time, after performing an ultrasound‐guided renal biopsy which showed segmental proliferation of the mesangial and endothelial cells, the diagnosis of FSGS was made. So, he was treated with pulse corticosteroid therapy (methylprednisolone 10 mg/kg/day) for three days, and after discharge, he underwent treatment with prednisolone with a dosage of 15 mg/day that was tapered by following weeks.

He also had a history of subclinical hypothyroidism. He denied a history of alcohol consumption or smoking; his family history was noncontributory to kidney or autoimmune diseases.

The patient's vital signs at the time of admission were as follows: temperature 36.9°C, pulse rate 81 beats/minute, blood pressure 131/82 mm Hg, respiratory rate 17 breaths/minute, and oxygen saturation 98% on room air.

On examination, two non‐tender, poorly mobile masses of 1.5 × 1 cm and 1.5 × 0.5 cm were located, respectively, in the right and left submandibular region of the neck with normal overlying skin. He had a 3+ pitting edema in both lower extremities. The remainder of the examination was unremarkable.

A chest radiograph showed no abnormal opacities, cavitations, or perihilar lymph nodes.

Laboratory findings of the patient are summarized in Table 1.

**TABLE 1:** Clinical laboratory findings.

WBC^1^	4200	cells/μL	Sodium	139	mEq/L	TSH^2^	8.28	μIU/mL
Neutrophil	52.2	%	Potassium	4.9	mEq/L	fT4^3^	4.27	ng/dL
Lymphocyte	35.5	%	Chloride	102	mEq/L	LDH^4^	715	IU/L
Red blood cell	5.7 × 10^6^	/μL	Calcium	7.7	mg/dL	ALP^5^	585	IU/L
Hemoglobin	11.7	g/dL	Phosphorus	4.7	mg/dL	ALT^6^	17	IU/L
Hematocrit	46	%	Albumin	1.7	g/dL	AST^7^	34	U/L
Platelet	47.2 × 10^4^	/μL				ESR^8^	90	mm/hour

Abbreviations: ALP, alkaline phosphatase; ALT, alanine aminotransferase; AST, aspartate aminotransferase; ESR, erythrocyte sedimentation rate; fT4, free thyroxine; LDH, lactic dehydrogenase; TSH, thyroid stimulating hormone; WBC, white blood cell.

24‐hour urine volume, proteinuria, and creatinine were reported 1100 mL, 3100 mg/day, and 1.4 mg/dL, respectively.

Urinalysis showed a specific weight of 1.025, red blood cells (15 cells/μl), and 2–4 granular casts in the urine sediment.

The results of rheumatologic and communicable disease screening tests were negative and in the normal range. Ultrasound examination of the thyroid gland showed thyroid lobes and isthmus with normal size and parenchymal echogenicity with no sign of solid or cystic lesion.

Ultrasonography of lymph nodes showed evidence of multiple lymphadenopathies on both sides of the neck, the largest of them measuring about 15 × 7 mm in the right submandibular region and 12 × 5 mm in the left carotid sheath.

In the setting of high serum levels of lactate dehydrogenase (LDH) and erythrocyte sedimentation rate (ESR), metastases of unknown origin and lymphoma were the highest diagnostic priorities, so the excisional biopsy of the largest mass (15 × 7 mm) was performed.

**FIGURE 1:**
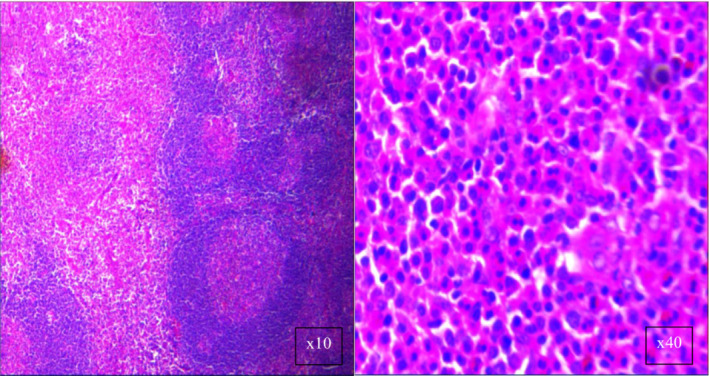
Pathology images of the excisional biopsy revealed lymph nodes with reactive follicles and prominent germinal centers.

Microscopic examination of stained sections (Figure 1) showed lymph nodes with reactive follicles and prominent germinal centers. High eosinophils levels and capillary hyperplasia were also evident. These histological characteristics were consistent with KD.

IgE serum levels were checked to make a definite diagnosis, which was reported 3425 g/L↑ (normal range, 0–200 g/L) with IgG, IgM, and IgA within normal ranges.

The treatment was started with oral prednisolone (15 mg/day) and mycophenolic acid (500 mg, bid), which resulted in a noticeable clinical improvement in the edemas and attenuation of proteinuria. The patient had a remarkable recovery and was discharged on day 10th of admission.

In a one‐year follow‐up, he was asymptomatic with lowered eosinophil count and no evidence of renal involvement relapse.

## 3 DISCUSSION

For the first time, KD was described in 1937 in China; since then, only around 400 cases have been reported worldwide.^10^

Classically, KD manifests with painless subcutaneous unilateral masses localized predominantly in the head and neck region.^11^ There are also several reported cases of subcutaneous masses in the groin, axilla, epicranium, oral cavity, nasal sinuses, auricle, orbit, eyelid, and inner canthus.^6^ In our case, the subcutaneous masses were located on both sides of the neck.

KD may be systemic and involve different organs, particularly kidneys.^2^ Renal involvement in KD is not uncommon,^5^ as approximately 10 to 60 percent of patients experience concomitant nephrotic syndrome^11^ that might be concurrent with mass lesions onset or even months or years afterward.^2^ Renal involvement in KD may present as FSGS, membrane glomerulonephritis, diffuse proliferative glomerulonephritis, minimal change disease, and mesangial proliferative glomerulonephritis.^12^ In our case, FSGS was the result of renal involvement.

The pathophysiology of KD and its related renal involvement remains unknown.^5^ Previous studies have suggested that KD may be associated with endocrine disorders, infections, and autoimmune diseases that trigger the IgE‐mediated type I hypersensitivity or induce a T‐cell‐mediated immune response, ultimately resulting in the deposition of eosinophils in involved tissue.^11^, ^13^ Moreover, genetics and sex hormones have been proposed to play a role in the pathogenesis of KD, as men are involved more than women.^14^

KD may be misdiagnosed clinically and not differentiated from T‐cell lymphomas, Hodgkin's lymphoma (28), uncommon infections, and sarcoidosis.^8^

The diagnosis of KD is based on history and examination, laboratory findings, and histological studies.^15^

The main histopathological findings of KD consist of increased eosinophils, follicular hyperplasia, and germinal centers containing IgE in obtained histologic specimens of the masses.^7^

Additionally, the position and size of KD‐induced masses can be determined by imaging studies, including computerized tomography, ultrasound, and magnetic resonance imaging which also makes surgical management easier.^3^ However, radiologic examinations have low specificity and may have similar findings with other benign and malignant conditions.^3^ Nevertheless, we are unable to make a definitive KD diagnosis until the excisional surgery and histological examinations of the masses have not been performed.^16^

Angiolymphoid hyperplasia with eosinophilia (ALHE) is another differential diagnosis of KD that has clinical and histopathological similarities with KD and may be confused with it.^17^ ALHE is a malignant disorder of vascular endothelial tissue that generally affects young women and manifests as solitary subcutaneous lesions.^17^, ^18^ However, elevated serum IgE levels, peripheral eosinophilia, and lymphadenopathy are rare in ALHE, which can solve the diagnostic challenge and differentiate it from Kimura disease.^17^, ^18^

There is no unique standard therapeutic approach for the management of KD.^6^ The majority of patients with renal impairment properly respond to oral corticosteroid therapy,^19^ but the relapse of renal manifestations after corticosteroid therapy is not uncommon.^20^ In this regard, for some cases with relapse after the termination of corticosteroid therapy, corticosteroids combined with immunosuppressive therapy, such as cyclosporine and cyclophosphamide, have been recommended.^21^, ^22^, ^23^ Cyclosporine may play a critical role in the remission of KD by suppressing the activity of the T‐cell cytokines.^24^

In addition to medical therapy, surgery, radiation, and chemotherapy have been recommended.^5^ In younger patients with localized lesions, surgical excision is typically the first‐line choice for treatment and diagnostic purposes.^2^ Furthermore, nephrotic syndrome relapse might be suppressed with the early excision of the KD masses.^25^

Radiation therapy has also been administered for patients who do not respond to corticosteroid therapy. On the contrary, irradiation can decrease the risk of the long‐term side effects of corticosteroids.^26^

In our case, the renal involvement of KD responded well to prednisolone with no subsequent relapse.

## 4 CONCLUSIONS

In conclusion, KD is a rare condition with challenging diagnosis and treatment for nephrologists and other clinicians because it may be confused with other disorders, such as infections and neoplastic diseases. Understanding the KD helps clinicians to make more appropriate diagnostic and management decisions for patients.

## AUTHOR CONTRIBUTIONS

**roya Hemayati:** Writing – original draft; writing – review and editing. **Fariba Binesh:** Investigation; writing – original draft. **Mohammad Pashmchi:** Writing – original draft; writing – review and editing. **Seyyed Mohammad Kazem Mousavi Anary:** Data curation. **Mohammad Mohammadi:** Conceptualization; data curation; investigation; project administration; supervision; visualization; writing – original draft; writing – review and editing.

## FUNDING INFORMATION

This study received no grant from any sources.

## CONFLICT OF INTEREST STATEMENT

The authors declare that they have no competing interests.

## ETHICAL APPROVAL

The current study was approved by the ethics committee of Shahid Sadoughi University of Medical Sciences, Yazd, Iran, and was performed in accordance with the ethical standards of the Institute Ethics Committee and the 1964 Helsinki declaration.

## INFORMED CONSENT

Written informed consent was obtained from the patient's parents for the publication of this case report.

## Data Availability

The data that support the present study are available from the corresponding author upon reasonable request.
